# MinION, a portable long-read sequencer, enables rapid vaginal microbiota analysis in a clinical setting

**DOI:** 10.1186/s12920-022-01218-8

**Published:** 2022-03-25

**Authors:** Shinnosuke Komiya, Yoshiyuki Matsuo, So Nakagawa, Yoshiharu Morimoto, Kirill Kryukov, Hidetaka Okada, Kiichi Hirota

**Affiliations:** 1 Department of Obstetrics and Gynecology, Kansai Medical University Graduate School of Medicine, Osaka, Japan; 2 HORAC Grand Front Osaka Clinic, Osaka, Japan; 3 Department of Human Stress Response Science, Institute of Biomedical Science, Kansai Medical University, Osaka, Japan; 4 Department of Molecular Life Science, Tokai University School of Medicine, Kanagawa, Japan; 5 Department of Informatics, National Institute of Genetics, Shizuoka, Japan

**Keywords:** 16S rRNA, Bacterial vaginosis, Long-read sequencer, MinION, Nanopore

## Abstract

**Background:**

It has been suggested that the local microbiota in the reproductive organs is relevant to women's health and may also affect pregnancy outcomes. Analysis of partial 16S ribosomal RNA (rRNA) gene sequences generated by short-read sequencers has been used to identify vaginal and endometrial microbiota, but it requires a long time to obtain the results, making it unsuitable for rapid bacterial identification from a small specimen amount in a clinical context.

**Methods:**

We developed a simple workflow using the nanopore sequencer MinION that allows high-resolution and rapid differentiation of vaginal microbiota. Vaginal samples collected from 18 participants were subjected to DNA extraction and full-length 16S rRNA gene sequencing with MinION.

**Results:**

The principal coordinate analysis showed no differences in the bacterial compositions regardless of the sample collection method. The analysis of vaginal microbiota could be completed with a total analysis time of approximately four hours, allowing same-day results. Taxonomic profiling by MinION sequencing revealed relatively low diversity of the vaginal bacterial community, identifying the prevailing *Lactobacillus* species and several causative agents of bacterial vaginosis.

**Conclusions:**

Full-length 16S rRNA gene sequencing analysis with MinION provides a rapid means for identifying vaginal bacteria with higher resolution. Species-level profiling of human vaginal microbiota by MinION sequencing can allow the analysis of associations with conditions such as genital infections, endometritis, and threatened miscarriage.

**Supplementary Information:**

The online version contains supplementary material available at 10.1186/s12920-022-01218-8.

## Background

Bacterial vaginosis (BV) is one of the most common gynecological disorders among women of reproductive age [1]. It is caused by disruption of the vaginal microbiota due to various factors such as psychological stress, poor physical condition, menstrual cycle, and pregnancy. It has relatively mild subjective symptoms, such as abnormal vaginal discharge (odor, color, amount of discharge) and itching, but there are cases of unrecognized infection and recurrent infections without significant symptoms [1]. BV has been clinically reported to cause adverse reproductive outcomes, including sexually transmitted infections, genitourinary viral infections such as HIV-1/2, HPV, and HSV-2 [2], and preterm delivery [3].

Currently, Amsel’s criteria based on clinical outcomes [4] and the Nugent score using Gram stain findings [5] are used as diagnostic criteria for BV. These methods are easy to use, but since they are based on the subjective opinion of the physician, the diagnosis largely depends on the skill of the examiner [6]. Bacterial culture of vaginal discharge is also a standard method to investigate the vaginal microbiota, but some bacteria are difficult to culture. Moreover, in routine gynecological practice, vaginal discharge is not sampled unless patients have symptoms suggestive of vaginosis. Therefore, accurately assessing the vaginal microbiota has been difficult with these regular clinical methods. Due to the lack of a reliable methodology, the association of BV with other vaginal disorders has not been fully elucidated. Until recently, a more detailed analysis of vaginal microbiota in asymptomatic women was considered excessive and might not be clinically relevant. Given that BV has been implicated in women's health outcomes in various ways, it is crucial to develop a rapid and accurate means for vaginal microbiome profiling with higher sensitivity. The increased risk of various infections, infertility, miscarriage and preterm birth due to BV has been shown to be impacted by the species that make up the vaginal microbiota [1–3]. If abnormal vaginal microbiota is identified with persistent cervical lesions, the dysbiosis should be treated even in the absence of symptoms. In this context, accurate representation of the vaginal microbiota will allow for more appropriate medical treatment, and it will potentially be beneficial for the diagnosis and prevention of gynecological diseases.

16S ribosomal RNA (rRNA) gene sequencing analysis using next-generation sequencers can be used to identify bacteria without culture in a manner not dependent on subjective assessment by the examiner. Previous studies have shown that the vaginal microbiota of healthy women is classified into community state type (CST) I to V [7]. CST is defined by the dominant bacteria as follows: CST I: *Lactobacillus crispatus*, CST II: *Lactobacillus gasseri*, CST III: *Lactobacillus iners*, CST IV: lack of *Lactobacillus* spp., and CST V: *Lactobacillus jensenni*. These narrow definitions mean that undiagnosed cases of vaginal dysbiosis may be present. This new classification of the vaginal microbiota has been clarified by performing sequencing analysis on cases with no clinical symptoms. Because a deeper and more accurate understanding of the vaginal microbiota may provide new clinical insights, we should demonstrate that 16S rRNA analysis is quick and easy to perform in a broad range of contexts in clinical practice.

The conventional short-read sequencing, such as the Illumina MiSeq technology, has been utilized as the standard method for the 16S rRNA gene-based analysis. The MinION sequencer from Oxford Nanopore Technologies is a newly developed sequencing platform. The MinION sequencer detects the signal of a DNA nucleotide that passes through a nanopore arranged on a flow cell [8]. It has no theoretical read length limit and generates long sequencing reads that cover the full length of the 16S rRNA gene. Accumulating evidence, including our previous study, demonstrates that MinION long-read sequencing provides comparable results to those obtained using highly accurate short-read sequencing data in a variety of biological samples [9–11]. Moreover, full-length 16S rRNA gene analysis via MinION has proven to have higher taxonomic resolution than short-read sequencing, offering a reliable means of profiling bacterial communities [11–13].

Here, we constructed an in-hospital vaginal microbiota-analyzing workflow using MinION. This feasibility study aims to demonstrate for the first time that MinION can provide a rapid and reliable means for an accurate representation of the vaginal microbiota.

## Methods

### Recruitment of participants

From April 2019 to May 2020, participants who met all of the following enrollment criteria were recruited: (1) primary infertility patients attending HORAC Grand Front Osaka Clinic in Osaka, Japan; (2) premenopausal Japanese women who were not pregnant; (3) no clinical symptoms strongly suggestive of BV; (4) scheduled hormone replacement embryo transfer cycle with frozen-thawed blastocyst; and (5) those who provided written consent. The exclusion criteria were as follows: (1) known history of HIV-1/2, hepatitis B/C, syphilis, or genital chlamydia infection; (2) known history of diabetes; (3) known history of cervical/vaginal surgery; (4) known history of intrauterine device use; and (5) known history of antibiotic, steroid, or vaginal suppository use within 2 weeks. In addition to collecting vaginal samples, details of age, body mass index, and basal hormone levels (anti-Mullerian hormone, luteinizing hormone, and follicle-stimulating hormone on days 2–4 of the menstrual cycle), medical history, and infertility treatment history were collected from the medical records. All vaginal samples were collected on the day of embryo transfer.

### Nugent score

Vaginal smears were analyzed by two pathologists affiliated with an external laboratory, and the Nugent score was calculated based on microscopic findings: the numbers of *Lactobacillus* (scored as 0 to 4), *Gardnerella* (scored as 0 to 4), and *Mobiluncus* (scored as 0 to 2). A total score of 0 to 3 was diagnosed as representative of healthy vaginal microbiota, 4 to 6 as an intermediate group, and 7 or more as BV [5]. Inappropriate samples with defective specimen collection were diagnosed as indeterminate. If BV was diagnosed based on the Nugent score, our policy was to treat it using antibiotics.

### Vaginal sample collection method

Vaginal lavage samples were collected from 18 participants; from four participants, additional swab samples were collected with written consent. When both lavage and swab samples were collected, the swab samples were collected first. For the lavage method, the inside of the vagina was washed with 10 ml of sterile saline, and at least 2 ml was collected using a sterile syringe. Collected lavage samples were stored at − 30 °C until DNA extraction. For the swab method, OMNIgene vaginal kit (OMR-130; DNA Genotek Inc., Ottawa, Canada) was used to infiltrate vaginal mucus from the uterovaginal region, and the swab tip was stored in the preservative solution of the sampling kit. Swab samples were stored at room temperature until DNA extraction in accordance with the manufacturer’s instructions.

### Sequencing sample preparation

DNA was extracted from 22 human vaginal samples using the QIAamp UCP Pathogen Mini Kit (QIAGEN, Venlo, Netherlands) following the manufacturer’s instructions. Briefly, samples were subjected to mechanical cell lysis by bead-beating, and DNA was isolated using silica membrane-based spin columns. After extraction, the DNA content was measured using a NanoDrop 1000 Spectrophotometer (Thermo Fisher Scientific, MA, USA), and the concentrations of extracted DNA from the swab and lavage samples were 6.1–32.3 ng/µl and 53.1–1092.4 ng/µl, respectively.

### MinION sequencing

A detailed protocol is available at protocols.io (https://dx.doi.org/10.17504/protocols.io.bwr5pd86). Amplification of 16S rRNA genes was performed with slight modifications according to the previously published protocol [11]. For amplification of the V1–9 region of the 16S rRNA gene, a forward primer (S-D-Bact-0008-c-S-20) with the anchor sequence 5′-TTTCTGTTGGTGCTGATATTGCAGRGTTYGATYMTGGCTCAG-3′ and a reverse primer with the anchor sequence 5′-ACTTGCCTGTCGCTCTATCTTCCGGYTACCTTGTTACGACTT-3′ were used as inner primers. For amplification of the V3–4 region, 341F with the anchor sequence 5′-TTTCTGTTGGTGCTGATATTGCCCTACGGGNGGCWGCAG-3′ and 806R with the anchor sequence 5′-ACTTGCCTGTCGCTCTATCTTCGGACTACHVGGGTWTCTAAT-3′ were used as inner primers. PCR amplification of the 16S rRNA gene was conducted using the KAPA2G Robust HotStart ReadyMix PCR Kit (Kapa Biosystems, MA, USA) with an inner primer pair (200 nM for each) in a total volume of 25 μl. Amplification was performed under the following PCR conditions: initial denaturation at 95 °C for 3 min; 30 cycles of 15 s at 95 °C, 15 s at 55 °C, and 30 s at 72 °C. The amplification products were confirmed using 1% agarose gel electrophoresis (1 × TAE buffer) to be approximately 1600 base pairs (V1–9 region) and 400 base pairs (V3–4 region), which are the expected amplification sizes. The resulting amplicons were subjected to the second PCR with the barcoded outer primers from PCR Barcoding Kit (SQK-PBK004; Oxford Nanopore Technologies, Oxford, UK) under the following PCR conditions: initial denaturation at 95 °C for 3 min; 10 cycles of 15 s at 95 °C, 15 s at 62 °C, and 30 s at 72 °C. After cleaning up the PCR products using AMPure XP (Beckman Coulter, CA, USA), a total of 100 fmoles (10 µl) of purified DNA was incubated with 1 µl of Rapid Adapter at room temperature for 5 min. The prepared DNA library (total of 11 µl) was mixed with 34 µl of Sequencing Buffer, 25.5 µl of Loading Beads, and 4.5 µl of water. The final adjusted sample was loaded into the flow cell R.9.4.1 (FLO-MIN106; Oxford Nanopore Technologies Ltd.) and sequenced on the MinION Mk1B, which was connected to a personal computer. In this study, four to six samples were loaded at one time and each sequencing session lasted about 90 min. DNA sequencing data were acquired in FAST5 format using MINKNOW software ver. 1.11.5 (Oxford Nanopore Technologies Ltd.).

### Bioinformatic analysis workflow

We performed the bioinformatic analysis using a pipeline of multiple programs built in accordance with our previous reports [14, 15]. An overview is given below.

1. Computer: Apple iMac 27-inch, Late 2015 (OS, macOS 10.14.6; CPU, 3.3 GHz Intel Core i5-6600; memory, 16 GB)
2. GUPPY software ver. 3.1.5 (Oxford Nanopore Technologies Ltd.): basecalling and demultiplexing with the following settings: Fast basecalling model, Barcode Kits_SQK-PBK004, trim_barcodes = on. Called reads (FASTQ format) were filtered by quality to generate pass reads with a minimum quality score of 7.
3. SeqKit software ver. 0.10.0 [16]: extraction of a read length of 1300 to 1950 bp for the V1–9 region and 350–600 bp for the V3–4 region, based on the 16S rRNA length registered in the SILVA rRNA database ver. 132 (https://www.arb-silva.de/) [17].
4. TANTAN program ver. 18 [18]: removing simple repeat sequences.
5. Minimap2 program ver. 2.14 [19]: eliminating human genome information (Human Genome Assembly GRCh38, https://www.ncbi.nlm.nih.gov/assembly/GCF_000001405.26/) and matching each genome read to the 5850 representative bacterial genome sequences (Additional file 1: Table S1) stored in the GenomeSync database (http://genomesync.org).
6. In-house Perl scripts (Genome Search Toolkit, http://kirill-kryukov.com/study/tools/gstk/): selecting the species with the highest Minimap2 score and determining the taxa based on the NCBI taxonomy database.
7. Krona software version 2.7 [20]: visualizing the frequency of detected bacterial species in a given sample.

### Statistical analysis

Principal coordinate analysis was performed to represent the microbial diversity using the UniFrac distance metric [21, 22]. The results were visualized with R version 4.0.2.

## Results

### Characteristics of participants

Our study consisted of 18 Japanese, ranging from 30 to 43 years of age, with a median age of 36.5. All participants had primary infertility with normal menstrual cycles, along with no underlying medical conditions (Table 1).

**Table 1:** Participants’ background

Number	Age (y.o.)	AMH (ng/mL)	LH (mIU/mL)	FSH (mIU/mL)	BMI (kg/m^2^)	Nugent score
1	36	0.47	7.2	6.8	18.8	1
2	36	1.15	3.7	5.0	25.6	4
3	42	0.23	8.3	11.3	21.0	5
4	30	9.16	6.8	7.2	26.0	1
5	43	1.67	2.8	5.5	20.8	0
6	33	3.11	3.2	5.5	25.0	0
7	35	5.89	5.1	7.9	20.9	2
8	35	4.98	2.6	4.9	18.8	6
9	43	2.26	1.8	6.0	19.4	0
10	39	0.59	6.5	6.3	22.4	1
11	37	2.34	8.9	6.0	18.6	0
12	34	4.52	5.2	5.8	20.3	0
13	35	2.62	8.8	8.2	23.0	2
14	37	3.16	2.0	3.8	22.1	4
15	40	3.69	8.2	7.6	20.4	0
16	38	2.27	6.4	8.4	18.1	0
17	35	3.66	6.9	7.8	20.4	3
18	38	0.44	3.5	6.0	19.1	2

*AMH* anti-Mullerian hormone, *LH* luteinizing hormone, *FSH* follicle-stimulating hormone, *BMI* body mass index

### Validation of MinION-based 16S rRNA gene sequencing for the detection of vaginal bacteria

We developed a rapid and more consistent method for constructing 16S rRNA gene sequencing libraries from clinical specimens with a relatively low bacterial load. We modified our previously published method and utilized the two-step PCR strategy, where the first PCR amplifies the near full-length 16S rRNA genes, followed by the second PCR that extends the amplicons with barcodes and adapters for sequencing on the MinION platform. The efficacy of this approach was evaluated using a pre-characterized mock community sample comprising ten bacterial strains. The V1–9 region of the 16S rRNA gene was amplified and sequenced on the MinION. Three thousand reads were randomly selected and aligned to the reference database of 5850 representative bacterial genomes. All the ten bacterial genera were identified with almost complete accuracy (Additional file 2: Fig. S1a). Furthermore, except for *Bacillus* and *Escherichia* strains, the majority of MinION reads were correctly classified to the species level (Additional file 2: Fig. S1b), confirming the validity of our method with high taxonomic resolution. Even with the full-length 16S rRNA gene analysis, species-level resolution was not possible for *Bacillus* and *Escherichia* [11, 23, 24].

**Fig. 1:**
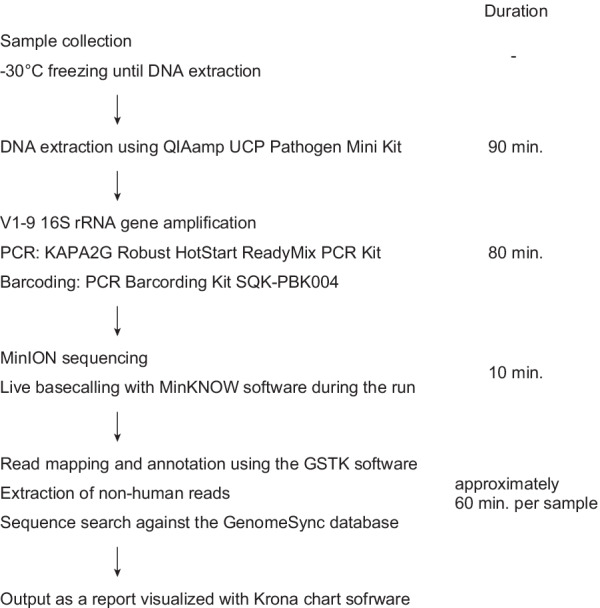
Workflow of 16S rRNA amplicon sequencing with the MinION platform and bioinformatic analysis. After collecting the vaginal sample, it should be stored in a freezer at − 30 °C until DNA extraction begins. Some swab collection kits should be stored at room temperature, in accordance with the manufacturer’s instructions. Sequencing libraries are generated by a four-primer PCR-based strategy; in the initial stages of PCR, the 16S rRNA gene is amplified with an inner primer pair. The PCR product is amplified with the outer primers and targeted to introduce identical barcode and tag sequences at both ends, allowing for the attachment of adapter molecules in a one-step reaction. The library is then loaded into a MinION connected to a personal computer. With our experimental method, sequencing runtime of 10 min is sufficient for 3000 reads to be obtained. A final report of the microbiota can be presented with a total analysis time of approximately 4 h

**Fig. 2:**
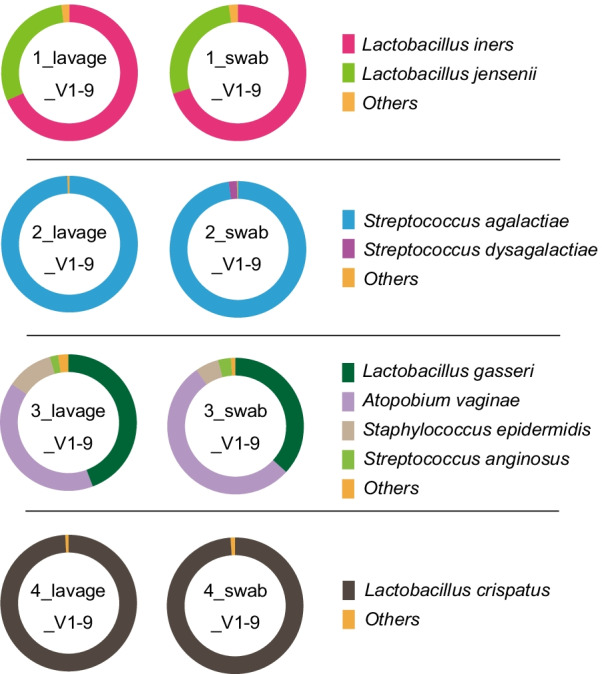
Taxonomic profiles comparing the different sampling method (lavage and swab) results of V1–9 16S rRNA MinION sequencing with 3000 randomly sampled reads after filtration. In the analytical algorithm, we assigned the bacterial name that showed the highest Minimap2 score for each read; bacteria with an assignment of less than 1% were included in “Others.”

**Fig. 3:**
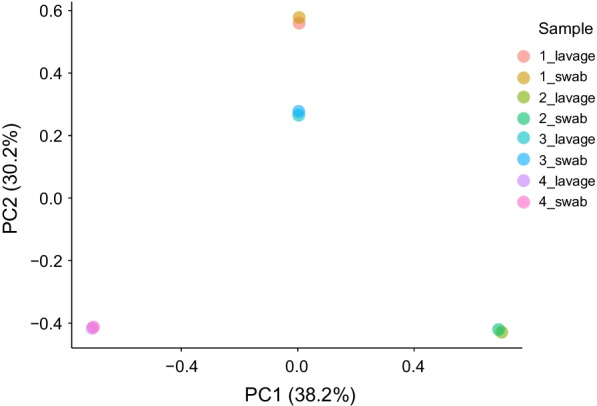
Principal coordinate analysis based on weighted UniFrac for vaginal microbiota with different sampling techniques. The coordinates of swab samples and lavage samples were nearly equivalent and did not show any effect of the vaginal specimen collection method in the 3000 filtered reads of V1–9 MinION sequencing

The composition of the vaginal microbiota was investigated by 16S rRNA gene amplicon sequencing on the MinION platform (Fig. 1). For subjects 1–4, lavage and swab samples were collected and 16S rRNA gene sequencing were performed to determine the effect of the sample collection method on the results of the microbiota analysis. The results obtained from 3000 processed reads in eight samples (1–4_lavage and 1–4_swab) showed that the sampling methods did not significantly impact the bacterial composition and the diversity indices (Fig. 2 and Additional file 3: Table S2) [25]. We performed principal coordinate analysis to assess the equivalence of the lavage and swab sampling methods, the results of which are shown in Fig. 3. Regardless of the sample collection method, the results of the analysis of the same subjects involved almost the same coordinates. Based on these observations, lavage samples collected with a less invasive procedure were used for subsequent analysis in this study.

### Characterization of vaginal microbiota

A total of 18 lavage samples were examined by 16S rRNA gene amplicon sequencing. The V1–9 MinION sequencing for almost 90 min yielded 15,836–119,745 reads and then filtered 10,688–103,212 reads satisfying the conditions of 1300–1950 bp in length and mean quality score > 7 (details in Table 2). Three thousand reads were randomly selected and mapped against the reference database for bacterial identification. The taxonomic diversity was evaluated by the Shannon diversity index. Taxonomic profiling by MinION sequencing revealed relatively low diversity, identifying the prevailing *Lactobacillus* spp. in the vaginal microbiota and other bacterial species such as *Gardnerella* and *Atopobium* frequently associated with BV (Additional file 3: Table S2). In parallel with 16S rRNA gene sequencing, samples were subjected to microscopic evaluation for Nugent scoring. Based on the Nugent score criteria, four out of 18 cases were in the intermediate group, but none of them were diagnosed as BV (Additional file 3: Table S2). The MinION sequencing data were also analyzed by an alternative bioinformatics tool. The FASTQ 16S workflow produced almost similar taxonomic profiles (Additional file 4: Table S3) and the bacterial compositions were comparable regardless of the program and database used.

**Table 2:** Statistics of MinION sequencing data

Sample	Pass reads				Filtered reads	
	No. of reads	Min (bp)	Avg (bp)	Max (bp)	No. of reads	Avg (bp)
1_lavage_V1-9	115,957	128	1434.3	4377	91,449 (78.9%)	1633.5
1_swab_V1-9	63,547	155	1078.0	4193	31,462 (49.5%)	1619.3
2_lavage_V1-9	33,721	158	1507.2	4486	28,718 (85.2%)	1607.0
2_swab_V1-9	53,584	145	1203.6	3746	33,294 (62.1%)	1613.6
3_lavage_V1-9	28,071	154	1396.3	4875	20,958 (74.7%)	1613.8
3_swab_V1-9	23,053	162	1521.0	3990	20,073 (87.1%)	1614.5
4_lavage_V1-9	30,603	155	1502.9	4977	25,756 (84.2%)	1639.5
4_swab_V1-9	37,597	161	1103.2	3383	19,588 (52.1%)	1644.9
5_lavage_V1-9	15,836	147	1346.1	3393	10,688 (67.5%)	1632.8
6_lavage_V1-9	38,020	144	1517.2	3787	32,255 (84.8%)	1642.4
7_lavage_V1-9	61,432	158	1218.8	5035	37,018 (60.3%)	1631.4
8_lavage_V1-9	52,071	111	1257.5	3344	33,906 (65.1%)	1603.2
9_lavage_V1-9	119,745	125	1530.5	4664	103,212 (86.2%)	1649.4
10_lavage_V1-9	102,333	150	1496.5	4980	84,028 (82.1%)	1650.2
11_lavage_V1-9	61,756	121	1541.3	3519	54,116 (87.6%)	1647.8
12_lavage_V1-9	101,755	120	1433.9	4759	77,756 (76.4%)	1635.8
13_lavage_V1-9	71,041	134	1437.5	4828	56,601 (79.7%)	1599.2
14_lavage_V1-9	42,629	159	1367.7	4981	31,447 (73.8%)	1629.0
15_lavage_V1-9	52,908	172	1452.9	3333	42,910 (81.1%)	1630.4
16_lavage_V1-9	52,230	153	1480.3	4345	43,549 (83.4%)	1633.0
17_lavage_V1-9	38,219	154	1478.3	3453	31,757 (83.1%)	1625.3
18_lavage_V1-9	28,886	185	1464.7	3328	23,816 (82.4%)	1631.8
5_lavage_V3-4	74,096	147	589.5	3038	29,505 (39.8%)	574.8
6_lavage_V3-4	83,983	141	586.1	2099	33,677 (40.1%)	571.8
9_lavage_V3-4	49,642	148	588.2	2275	21,614 (43.5%)	574.2
10_lavage_V3-4	65,561	139	587.2	2873	28,440 (43.4%)	574.5
11_lavage_V3-4	98,126	124	587.4	2568	40,485 (41.3%)	571.7
14_lavage_V3-4	79,631	127	584.9	1,849	35,009 (44.0%)	571.2

*Min* minimum read length, *Avg* average read length, *Max* maximum read length. The reads were filtered by size, retaining 1300–1950 bp sequences for the V1–9 region and 350–600 bp sequences for the V3–4 region

### Comparison of the results of sequence read number

**Fig. 4:**
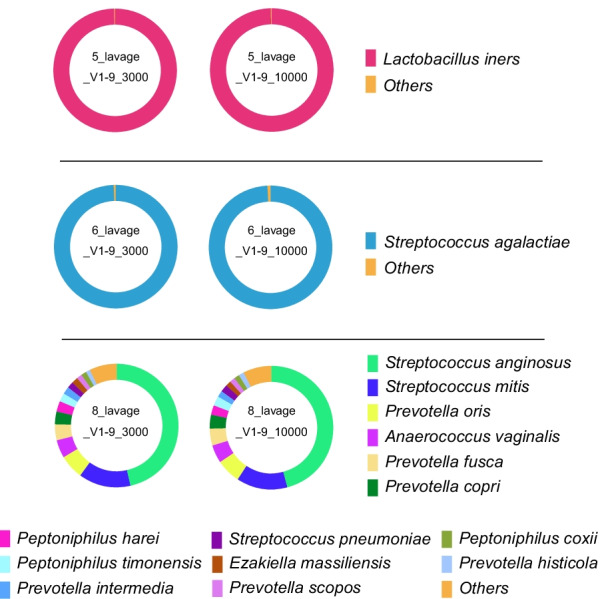
Taxonomic profiles comparing the results obtained for 3000 and 10,000 filtered reads of V1–9 MinION sequencing. In the analytical algorithm, we assigned the bacterial name that showed the highest Minimap2 score for each read; bacteria with an assignment of less than 1% were included in “Others.”

To assess the effect of read count on the results of the MinION sequencing, three samples that showed characteristic bacterial microbiota [5_lavage: *Lactobacillus iners* dominant (> 99%), 6_lavage: *Lactobacillus crispatus* dominant (> 99%), 8_lavage: BV like] were selected and their taxonomic profiles were compared after filtering for 3000 and 10,000 reads (Fig. 4). In all specimens, the results of vaginal microbiota analysis in MinION were similar for different numbers of reads.

### Comparison of the results of V1–9 and V3–4 16S rRNA gene sequencing

**Fig. 5:**
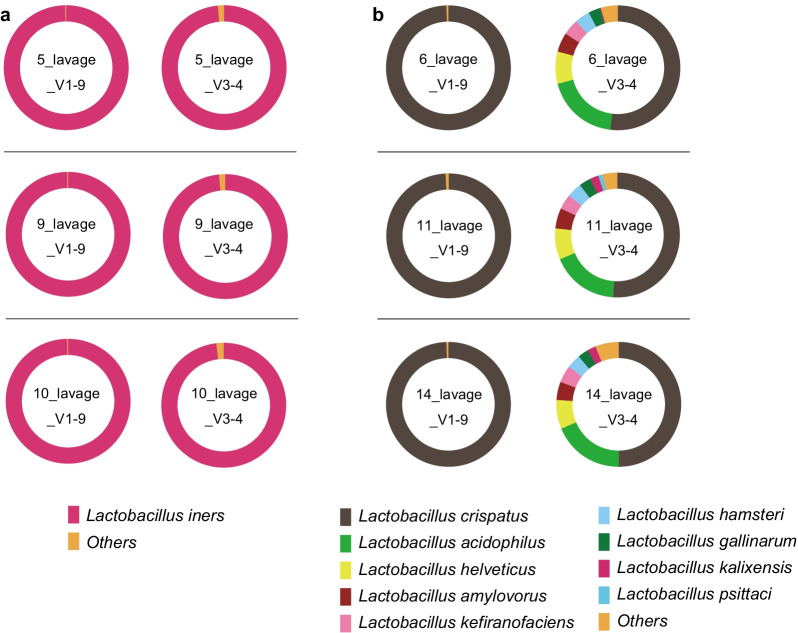
Taxonomic profiles comparing the results of V3–4 and V1–9 16S rRNA sequences using MinION in 3000 filtered reads. **a** In three cases, *Lactobacillus iners* was shown to constitute more than 99% of the bacteria by MinION analysis with the V1–9 region (Group I). **b** In three cases, *Lactobacillus crispatus* was shown to constitute more than 99% of the bacteria by MinION analysis with the V1–9 region (Group C)

For the taxonomic classification of bacteria, we compared the resolution of long-read (V1–9) and short-read (V3–4) 16S amplicon sequencing for six samples (sample numbers 5, 6, 9, 10, 11, and 14). Of these, for sample numbers 5, 9, and 10, *Lactobacillus iners* was confirmed to be present at a rate of more than 99% (Group I), while for sample numbers 6, 11, and 14, *Lactobacillus crispatus* was confirmed to be present at more than 99% (Group C) in the V1–9 16S rRNA analysis. The V3–4 region was amplified from the six samples and sequenced with MinION (Table 2). Three thousand V3–4 reads ranging in size from 300–650 bp were randomly extracted, and the classification results were compared with those obtained by the V1–9 full-length sequencing (Fig. 5). In Group I, *Lactobacillus iners* was classified as being present at a rate of more than 98% in the V3–4 S16 rRNA analysis, which was comparable to the results obtained by the V1–9 sequencing (Fig. 5a). In Group C, however, the V3–4 sequence alignment resulted in ambiguous identification of *Lactobacillus* species (Fig. 5b). The considerable number of V3–4 reads were not assigned to a specific taxon but they were allocated to multiple taxonomic bins representing potentially existing species. These ambiguous reads were not observed in V1–9 data.

## Discussion

The vagina is characterized by particularly low bacterial diversity compared with the human gut harboring a complex microbial community. Recent studies suggest the role of microbiota in the maintenance of vaginal health, and the characterization of the vaginal bacterial communities has increasingly been of interest [26]. Conventional methods, such as Amsel’s criteria, the Nugent score, and bacterial culture, are not suitable for an accurate bacterial identification. Next-generation sequencing has revolutionized the profiling of the bacterial microbiota. In short, the combination of metagenomic sequencing and bioinformatic technology has made it possible to more accurately assess the bacterial microbiota without the influence of uncertainties such as examiner subjectivity or the capturability of each bacterium [6].

The nanopore sequencer MinION enables real-time analysis of long DNA sequences and provides high-resolution taxonomic profiling of bacterial communities. Having adopted a minimally invasive sampling procedure combined with the long-read sequencing technology, we have established a standardized protocol applicable to vaginal specimens with a low bacterial load. Efficient disruption of bacterial cells by bead-beating and a rapid library construction with an optimized primer set enabled the successful identification of a broad range of bacterial species. The reliability of the method was confirmed by analyzing the pre-characterized mock community sample, where the majority of reads were correctly classified down to the species level. Given that the validity of the method has been verified, this study is the first to evaluate the feasibility of MinION sequencing for the characterization of the vaginal microbiota. For the most part, the 16S rRNA gene analysis appeared to correlate with the Nugent scoring to diagnose BV. Although no cases were diagnosed as BV by the Nugent scoring method, the 16S rRNA gene analysis using MinION had an ability to reveal the presence of BV-causing bacteria such as *Streptococcus*, *Atopobium*, *Prevotella*, and *Gardnerella* [7] in the group categorized as intermediate based on Nugent's criteria. In one case (14_lavage), the sample was categorized as intermediate by Nugent scoring, whereas MinION sequencing suggested a healthy microbiota composition only with *Lactobacillus*. While Nugent scoring is a subjective evaluation and depends on the acumen of the examiner, the results may indicate that the molecular method targeting the 16S rRNA gene could serve as a practical and reliable measure for the diagnosis of BV.

The conventional parallel-type short-read sequencer cannot yield reads covering the full length of the 16S rRNA gene, and the partial 16S rRNA gene sequencing has been insufficient to identify bacterial taxa at the species level. Benchmarking with the conventional sequencing method (e.g., Illumina MiSeq technology) demonstrated that the full-length 16S gene sequencing by MinION gives a better resolution for bacterial identification [11–13]. Consistently, we showed that the short read (V3–4) sequencing exhibited lower discriminatory power than the full-length (V1–9) sequencing in the profiling of the vaginal microbiota. The considerable number of V3–4 reads were prone to be misclassified and could not be assigned to a single species in the taxonomic classification. Some of these reads were evenly allocated to multiple taxonomic bins with identical similarity scores, representing potentially existing species. The numbers of such ambiguous reads were far smaller in the V1–9 data set. This suggests that assigning bacteria using the V1–9 region is more informative for determining the appropriate bacterial species than using only the V3–4 region, and long-read sequencing with MinION showed high identification accuracy in the characterization of vaginal microbiota.

The full-length 16S rRNA gene sequencing afforded by MinION has a potential to determine the correct microbial composition up to the species level. In vaginal microbiota analysis, it is important to be able to profile *Lactobacillus* species. It has been reported that species-specific characteristics of *Lactobacillus* are critical for the stability and maintenance of human vaginal microbiota [27, 28]. In particular, *Lactobacillus iners*-dominated community has been more associated with vaginal dysbiosis, suggesting its relevance in the pathophysiology of gynecological diseases. It has also been suggested that the implantation rate was decreased in groups with lower proportions of *Lactobacillus* species, and the degree of dominance of *Lactobacillus crispatus* could affect the pregnancy outcome [29]. Since the species-level taxonomic resolution is not attainable with the existing culture-dependent techniques or morphotype-based characterization, vaginal microbiota profiling by the MinION sequencing might provide a novel and feasible option to help improve the chances of pregnancy in the fertility treatment.

In terms of the usefulness of the MinION sequencer in gynecological practice, it offers the additional advantage of a fast turnaround time. Rapid identification of bacterial pathogens is crucial for the treatment of infectious diseases. It has been suggested that vaginal dysbiosis may have a connection with the incidence of sexually transmitted infections [30] and genital viral infections [31, 32] or cervical lesions [33]. Furthermore, the temporal dynamics of the vaginal microbiota has been reported, where the compositions of bacterial communities changed markedly during the menstrual cycle [34]. An efficient and rapid means of bacterial identification is required to detect the changes in the microbiota composition over such short periods of time. In contrast to the characterization of highly complex bacterial communities in the human gut, the analysis of vaginal microbiota of lower complexity would require a much smaller number of reads. A calculation using the median filtered reads predicted that 3000 reads would be accumulated in a sequence of approximately 8 min, indicating that the time required for vaginal microbiota analysis using MinION could be further reduced. The conventional parallel-type short-read sequencers require multiple samples to be processed in one batch when considering cost-effectiveness. On the other hand, a small number of samples can be processed with the MinION on a case-by-case basis, making the analysis economically feasible. In these contexts, the full-length 16S rRNA analysis using MinION could be a promising option for bacterial identification in a reasonable time frame for diagnostic purposes.

Having used MinION in our vaginal microbiota analysis workflow, we have seen four key benefits. (1) Convenience: The unit is extremely light, small, and portable, and sequencing can occur via a USB connection to an in-clinic PC. (2) Speed: Same day results are available with a total analysis time of approximately 4 h. (3) Economy: Analysis of a small number of samples can be performed without increasing the unit cost of the test, by using individual barcodes. (4) Functionality: Full-length 16S rRNA gene amplicon sequencing offers the high-resolution taxonomic analysis of the vaginal microbiota. Although this study focused on the vaginal microbiota, a similar workflow could be applied to many clinical areas in the future, and the benefits of MinION could make it easier for clinicians to successfully perform bacterial metagenome analysis. The metagenomic approach targeting the bacterial genetic markers can provide a universal diagnostic measure for the identification of inflammatory bacterial pathogens as well as for investigating tissue dysbiosis associated with human diseases [35]. In addition, future large-scale microbiota studies could lead to new clinical findings.

## Conclusions

In conclusion, our validation shows that the MinION long-read sequencer provides a low-cost, rapid workflow for identifying vaginal microbiota with higher resolution in a clinical setting. Detecting vaginal microbiota at the species level has the potential to identify risks to women’s health, as well as facilitating large-scale clinical studies in any medical field.

## Supplementary Information

**Additional file 1: Supplementary Table S1.** Representative bacterial genomes stored in the GenomeSync database.

**Additional file 2: Supplementary Fig. S1.** Taxonomic assignments of a mock community of 10 known bacterial species analyzed by MinION sequencing.

**Additional file 3: Supplementary Table S2.** Details of identified species with V1-9 16S rRNA analysis using MinION.

**Additional file 4: Supplementary Table S3.** Taxonomic profiles of the vaginal microbiota analyzed by FASTQ 16S workflow.

## Data Availability

Sequence data from this article have been deposited in the DDBJ DRA database (www.ddbj.nig.ac.jp/dra/index-e.html) under accession numbers DRR244979–DRR245006.

## Abbreviations

BV: Bacterial vaginosis
CST: Community state type
PCR: Polymerase chain reaction
rRNA: Ribosomal RNA
