# Supplementary material for: MinION, a portable long-read sequencer, enables rapid vaginal microbiota analysis in a clinical setting
Source: BMC Med Genomics. 2022 Mar 25;15:68. doi: 10.1186/s12920-022-01218-8 (PMC8953062; doi:10.1186/s12920-022-01218-8)
Supplement: Supplementary file 2 — Additional file 2: Supplementary Fig. S1. Taxonomic assignments of a mock community of 10 known bacterial species analyzed by MinION sequencing. [file 12920_2022_1218_MOESM2_ESM.pdf]

a

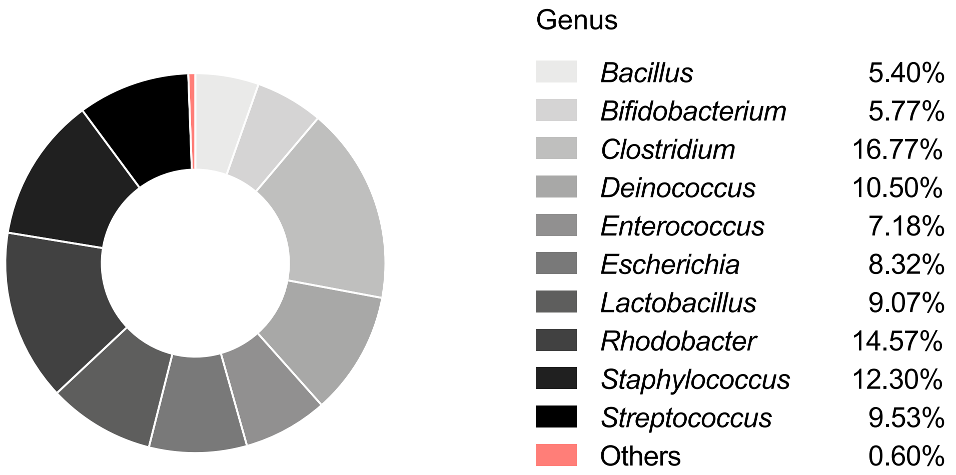

b

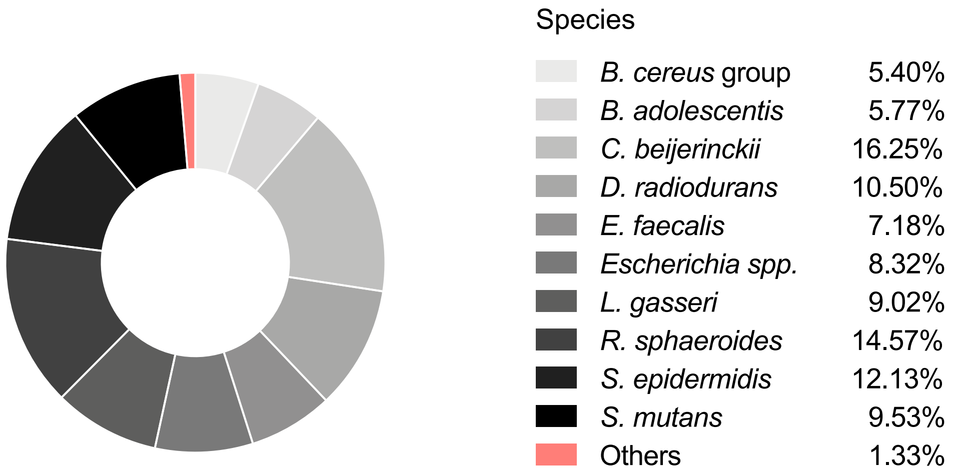

Supplementary Fig. S1 Taxonomic assignments of a mock community of 10 known bacterial species analyzed by MinION sequencing. The near full-length (V1-9) 16S rRNA genes were amplified from a mock community sample (ATCC 10 Strain Even Mix Genomic Material, MSA-1000), comprising the following bacterial strains: *Bacillus cereus*, *Bifidobacterium adolescentis*, *Clostridium beijerinckii*, *Deinococcus radiodurans*, *Enterococcus faecalis*, *Escherichia coli*, *Lactobacillus gasseri*, *Rhodobacter sphaeroides*, *Staphylococcus epidermidis*, and *Streptococcus mutans*. The resulting amplicons were sequenced on the MinION platform, and randomly sampled 3000 reads were used for taxonomic profiling. The relative abundances (%) of each taxon at the (a) genus and (b) species levels are shown. Misclassified (assigned to bacteria not present in the mock community) or unclassified reads are presented as "Others". Species-level discrimination is not possible for members of the *Bacillus cereus* group and *Escherichia* species, sharing high 16S rRNA gene sequence similarities within the genera.
